# Diagnosis and treatment of splenic torsion in children: preoperative thrombocytosis predicts splenic infarction

**DOI:** 10.1186/s12887-022-03484-y

**Published:** 2022-07-22

**Authors:** Zengmeng Wang, Chunhui Peng, Dongyang Wu, Kai Wang, Yajun Chen

**Affiliations:** General Surgery Department of Beijing Children’s Hospital, National Center for Children’s Health, Capital Medical University, 56# Nanlishi Road, Beijing, 100045 China

**Keywords:** Splenic torsion, Wandering spleen, Imaging, Thrombocytosis, Splenectomy, Pediatric

## Abstract

**Background:**

Pediatric splenic torsion is a rare entity, and the most common cause is wandering spleen. This study aimed to summarize our clinical experience in the diagnosis and surgical treatment pediatric patients with splenic torsion, and to use preoperative thrombocytosis as a preoperative predictive factor for splenic infarction.

**Methods:**

From January 1st, 2016 to December 31st, 2021, 6 children diagnosed as splenic torsion were included. All patients were surgically treated and followed up. The clinical data was collected including clinical presentations, laboratory tests, imaging results, surgical procedures, and prognosis. Clinical experience of diagnosis and surgical treatment were summarized.

**Results:**

There were 4 females and 2 males, with median age at surgery 102.6 (range 9.4–170.7) months. Abdominal pain and abdominal mass were the most common presentations. The diagnosis of splenic torsion depended on imaging studies, and adjacent organ involvement (gastric and pancreas torsion) was observed on contrast CT in one patient. Five patients were diagnosed as torsion of wandering spleen, and one was torsion of wandering accessory spleen. Emergent laparoscopic or open splenectomy was performed in all patients. Pathology revealed total splenic infarction in 4 patients, partial infarction in 1 patient, and viable spleen with congestion and hemorrhage in 1 patient. Preoperative platelet counts were elevated in all 4 patients with splenic infarction, but normal in the rest 2 with viable spleen. Postoperative transient portal vein branch thromboembolism occurred in one patient.

**Conclusions:**

Imaging modalities are crucial for the diagnosis of pediatric splenic torsion and adjacent organ involvement. Preoperative thrombocytosis may predict splenic infarction. Spleen preserving surgery should be seriously considered over splenectomy in patients with a viable spleen.

**Supplementary Information:**

The online version contains supplementary material available at 10.1186/s12887-022-03484-y.

## Background

Splenic torsion due to wandering spleen is rare, and no accurate incidence has been reported [1]. It accounts for less than 0.25% of all splenectomies, and has a bimodal distribution [2, 3]. About one third of all patients were children, and in adults it mainly affects women of reproductive age (20–40 years old) [4, 5]. Early diagnosis and timely surgical intervention are important to preserve the spleen, especially in children [6, 7]. In this rare entity, no preoperative predictive factor for splenic infarction has been proposed so far. In this study we summarize our clinical experience from a case series of 6 pediatric patients with splenic torsion, to add evidence of diagnosis and treatment in this rare entity, and we propose to use preoperative thrombocytosis as a simple predictive factor for splenic infarction. To our knowledge this is a relatively large case series of pediatric splenic torsion due to its rarity [4, 8].

## Methods

### Study design

From January 1st, 2016 to December 31st, 2021, 6 children diagnosed as splenic torsion due to wandering spleen were admitted into our general surgery department, and all 6 patients were included in this case series. The diagnoses of splenic torsion due to wandering spleen were confirmed by surgery. Clinical experience of diagnosis and surgical treatment were summarized.

### Data collection

Inpatient and outpatient medical records were thoroughly reviewed for data collection, including demographic characteristics, clinical presentations, laboratory tests, imaging results, surgical procedures, and prognosis. Follow ups were carried out by outpatient visits and phone interviews. Any recorded surgical complications were analyzed, and any post-splenectomy complications, such as overwhelming post-splenectomy infection, arterial and venous thrombotic events, and pulmonary hypertension, were checked during follow-ups.

### Statistical methods

Due to the limited case numbers, data were displayed by statistical descriptions, tables, and charts. IBM SPSS Statistics 26.0 was used to manage the data.

## Results

### Detailed information of all cases

There were 4 females and 2 males, with median age at surgery 102.6 (range 9.4–170.7) months. Detailed clinical information is demonstrated in Table 1. The final diagnoses were splenic torsion due to wandering spleen in 5 cases (case 1–5), and wandering accessory spleen torsion in 1 case (case 6).

**Table 1:** Detailed clinical information of 6 cases

No	Sex	Age (mon)	Clinical Presentation &Duration	Imaging (modality/spleen perfusion/spleen location)	Surgery (approach/degree of torsion)	Pathology	Spleen Size (cm)	Addition
1	F	18.0	abdominal pain&mass 10 days	U + contrast CT no perfusion left flank	OS 720°	total infarction	8*6*5	adhesion to omentum and ileum
2	F	143.9	abdominal pain&nausea 1 month	U + contrast MRI no perfusion pelvis	LS → OS 360°	total infarction &fibrosis	15*8*6	dense adhesion to descending colon causing conversion
3	M	153.3	abdominal pain&mass 12 months	U + contrast MRI reduced perfusion left flank	OS 360°	congestion &hemorrhage	16*9*8	failed to perform splenopexy due to splenomegaly
4	F	61.3	abdominal pain&fever 7 days	U + contrast MRI no perfusion pelvis	LS → OS 1800°	total infarction	11*7*5	intraoperative bleeding from the pedicle causing conversion
5	F	170.7	abdominal pain&vomiting 10 days	U + contrast CT partial perfusion pelvis	LS 270°	partial infarction; partial congestion &hemorrhage	17*14*10	gastric and pancreatic torsion; gastric varices; splenic vein thrombosis; postoperative portal vein thromboembolism
6	M	9.4	irritability &fever 5 days	U + contrast CT no perfusion of accessory spleen left flank	OS of accessory spleen 720°	total accessory spleen infarction	accessory spleen 8*5*3	torsion of wandering accessory spleen

*F* Female, *M* Male, *U* Ultrasonography, *CT* Computed tomography, *MRI* Magnetic resonance imaging, *OS* Open splenectomy, *LS* Laparoscopic splenectomy, →  = Conversion

### Clinical presentations and imaging features

The most common clinical presentations were abdominal pain/irritability (6/6) and abdominal mass (3/6). Other symptoms included fever, nausea, and vomiting. The symptom duration ranged from 5 days to 12 months. The diagnoses were established on imaging results, including ultrasonography, and more importantly, the contrast cross sectional imaging. Imaging results demonstrated no splenic perfusion in 4 patients (case 1, 2, 4, and 6), partial perfusion in 1 patient (case 5), and reduced perfusion in 1 patient (case 3). The wandering spleen/accessory spleen located in left flank in 3 patients and pelvis in the rest 3 patient.

**Fig. 1:**
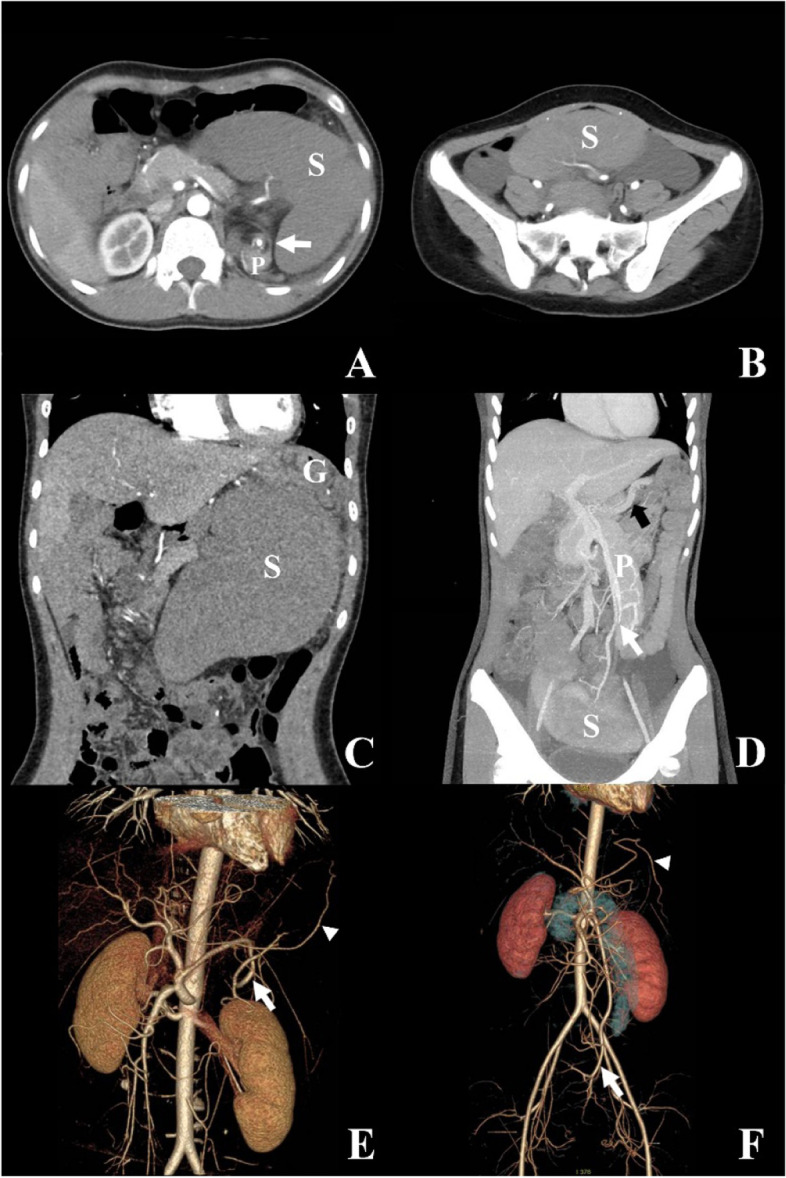
Comparison of two preoperative contrast CT results with 6 days interval in case 5, showing the spleen moving from left hypochondrium to the pelvis. A&B: The splenic enhancement was reduced in **A** and was partially enhanced in **B**. (S = spleen, *P* = pancreas, white arrow shows “Whirl sign” with enhanced splenic artery in the center and pancreatic tail involved.). C&D: Coronary views show normal location of gastric fundus in **C** (G = gastric fundus), splenic vein interruption in **D** (white arrow, indicating thrombosis) and engorged left gastric vein in **D** (black arrow). **E**&**F**: Artery reconstruction views show a splenic artery (white arrow) circling (indicating torsion) in **E** and running downward in **F**, and route change of gastro-epiploic artery (white triangle, indicating gastric volvulus)

In case 5, the first contrast CT on the day of admission showed a large spleen with reduced enhancement and splenic vein thrombosis. Torsion of the spleen was indicated by the whirl sign of the pedicle, with pancreatic tail involved (Fig. 1A). The spleen was at left hypochondrium (Fig. 1C). On artery reconstruction image the vascular route of splenic artery showed a circle which was consistent with the whirl sign, but the gastro-epiploic artery was normal (Fig. 1E). However, since the position of the spleen was generally normal, the whirl sign was missed initially, which caused delay in diagnosis. Until on the 6th day after admission, an abdominal mass appeared at lower abdomen, that wandering spleen with splenic torsion was suspected. A second contrast CT showed the spleen had moved into the pelvis with pedicle torsion (whirl sign, see supplementary video 1) and splenic vein thrombosis (Fig. 1D). The spleen was partially enhanced indicating partial perfusion (Fig. 1B). The torsion of wandering spleen also caused torsion/disposition of the pancreatic tail, and a mescenteroaxial gastric volvulus (Fig. 1D). The splenic artery and gastro-epiploic artery ran abnormally downward and was elongated (Fig. 1F). Engorged left gastric vein and gastro-epiploic vein, and gastric fundus varices were also noticed (Fig. 1D).

**Fig. 2:**
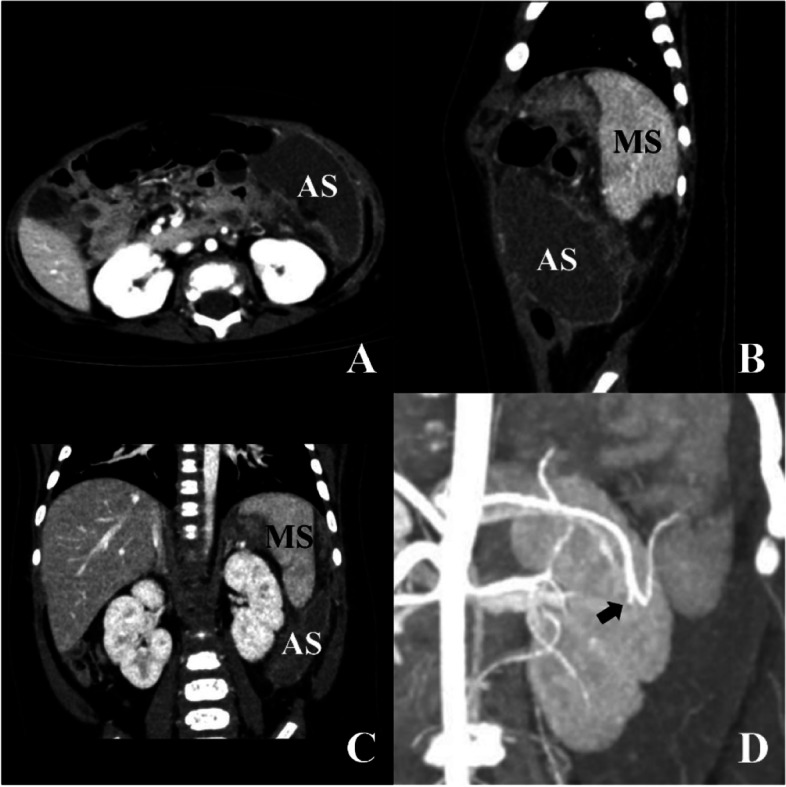
Preoperative contrast CT result of case 6, showing accessory splenic torsion with infarction (AS = accessory spleen, MS = main spleen). Rim sign (enhancement of the capsule with non-enhancement of the parenchyma) of the accessory spleen was obvious, indicating infarction in **A** (cross sectional view), **B** (sagittal view) and **C** (coronary view). The main spleen was at normal location with normal perfusion in **B** and **C**. The accessory splenic artery originated from the main splenic artery and was interrupted soon after branching in **D** (black arrow)

In case 6, the contrast CT showed a normal located main spleen with an accessory spleen of almost equal size, and both receiving blood supply from splenic artery. The artery of the accessory spleen was interrupted soon after branching (Fig. 2). The main spleen was in normal position with normal perfusion. The accessory spleen was in left flank with no perfusion and rim sign, which was consistent with torsion and infarction [9, 10].

### Surgery, pathology, and the association with preoperative platelet count

#### Surgical results

**Fig. 3:**
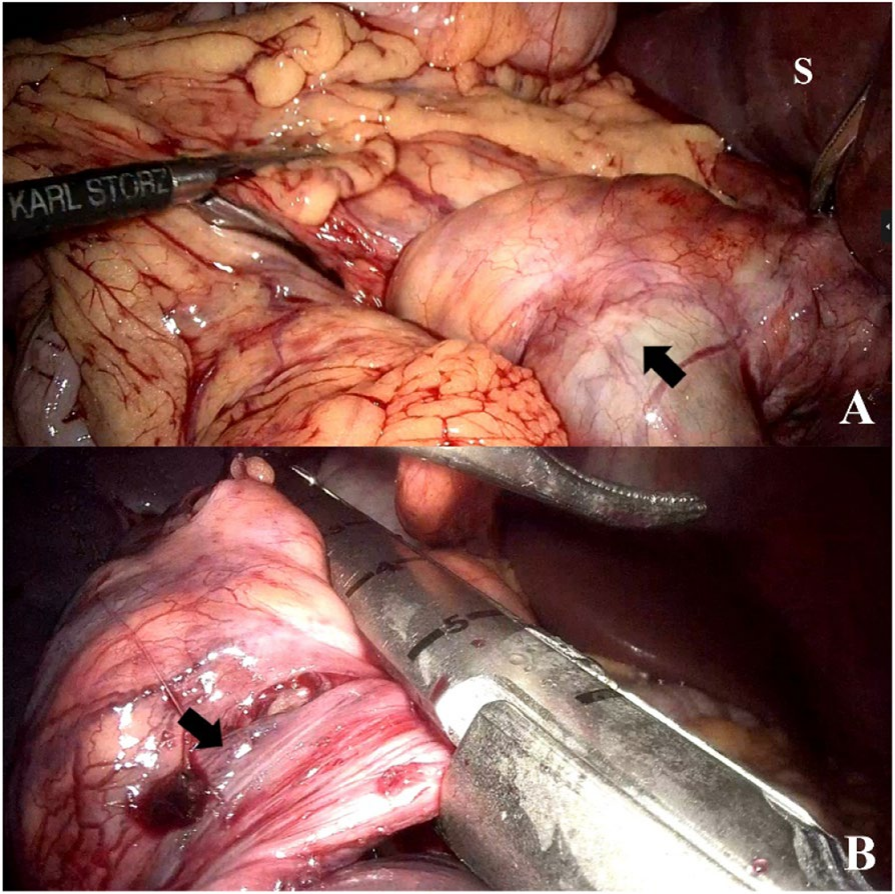
Intraoperative laparoscopic view of case 5. **A**: Splenic pedicle torsion (black arrow) with engorged splenic vein (S = spleen). **B**: Splenectomy after detorsion using an Endo-GIA linear stapler, resecting the thrombosed vascular pedicle as much as possible and avoiding injury to the pancreas

Laparoscopic or open splenectomy was performed in all patients. There were two conversions during laparoscopic procedures, due to dense adhesions to descending colon in case 2 and pedicle vascular bleeding in case 4. In case 3 the spleen was viable after detorsion but large, causing difficulty to perform splenopexy with retroperitoneal pouch (unable to create a retroperitoneal pouch large enough, hard to manipulate the large spleen under laparoscope causing iatrogenic spleen laceration and bleeding, no suitable mesh, and long operating time), and was finally resected. In case 5, considering the splenic vein thrombosis, though the spleen was partially viable, laparoscopic splenectomy was performed using Endo-GIA linear stapler (Fig. 3). The elongated splenic pedicle was resected as much as possible in order to remove the thrombus, without injuring the pancreatic tail. After resection, the stomach (see supplementary video 2) and pancreatic tail was replaced to normal position.

### The association of pathology and preoperative platelet count

Pathological results were consistent with preoperative imaging perfusion results, that splenic infarction corresponded with no perfusion in 4 patients (case 1,2,4,6), and splenic congestion and hemorrhage corresponded with reduced perfusion in 2 patients (case3,5).

**Fig. 4:**
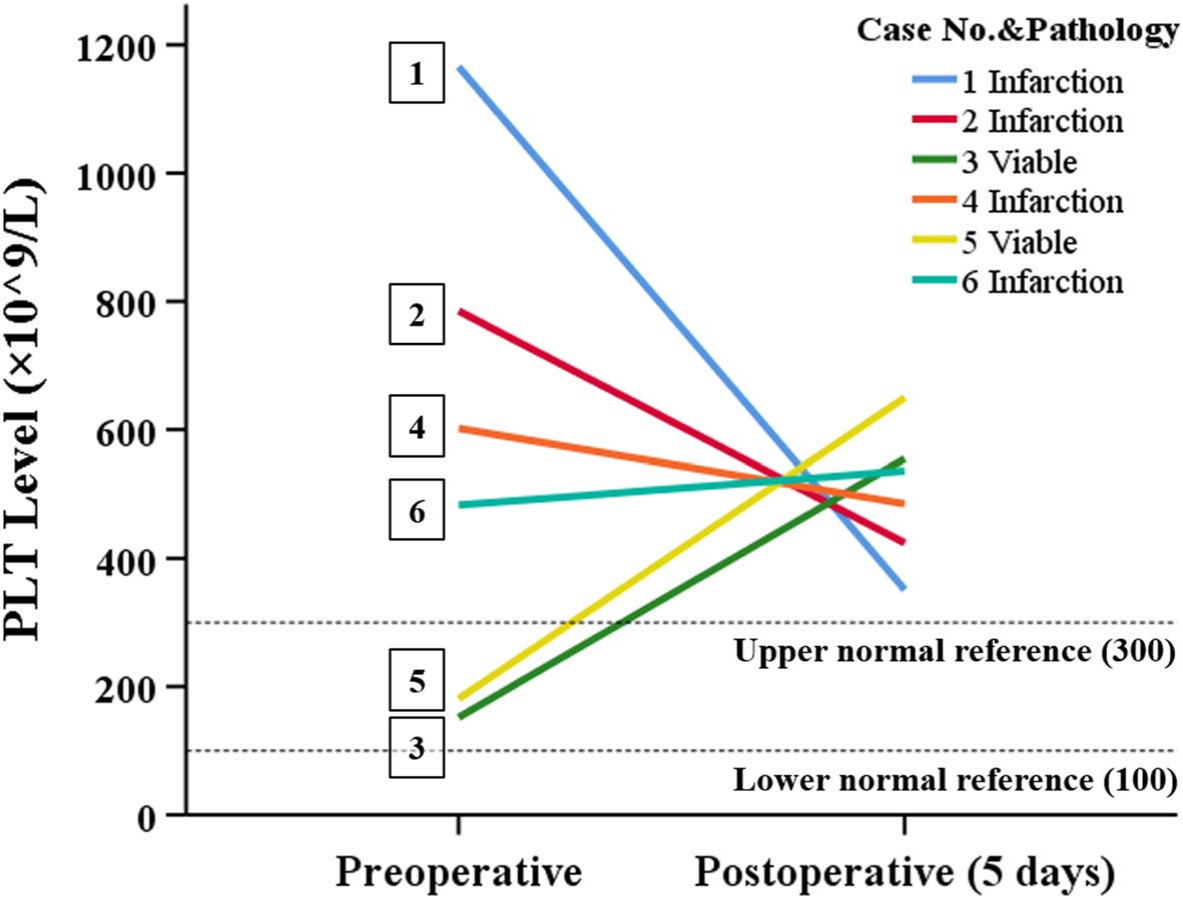
The association of platelet counts and splenic viability in all cases. All patients with preoperative thrombocytosis were confirmed to have splenic infarction

The pre- and postoperative (1st and 5th day) blood routine test results of all patients are showed in Table 2. The changes of platelet counts of all patients are showed in Fig. 4. All 4 patients with splenic infarction had preoperative thrombocytosis, and the rest 2 patients with viable spleen had normal preoperative platelet counts. All patients had thrombocytosis on the 5th postoperative day.

**Table 2:** The pre- and postoperative blood routine test results of all patients^a^

	Preoperative			Postoperative (1st day)			Postoperative (5th day)		
	WBC (× 10^9^/L)	Hb (g/L)	PLT (× 10^9^/L)	WBC (× 10^9^/L)	Hb (g/L)	PLT (× 10^9^/L)	WBC (× 10^9^/L)	Hb (g/L)	PLT (× 10^9^/L)
Case 1	21.77	120	1166	21.72	109	946	12.12	115	351
Case 2	7.37	115	785	9.88	106	505	5.15	116	424
Case 3	14.5	136	152	16.03	134	297	8.10	151	555
Case 4	8.59	94	602	6.48	100	675	7.92	101	485
Case 5	7.26	130	181	8.99	98	228	4.74	98	650
Case 6	9.40	92	483	8.08	136	506	18.74	133	536

^a^No blood transfusion in all patients

### Prognosis

All patients were advised to accept vaccines against encapsuled bacteria (multi-valent pneumococcal vaccine, H influenzae type b vaccine, and the meningococcal vaccine) 2 weeks after surgery. The median time of postoperative follow-up was 43.4 (range 9.4–74.1) months. In case 4, two days after laparoscopic splenectomy a thrombus (0.6*0.5*0.5 cm) was detected in the sagittal portion of left portal vein (Rex recessus) by ultrasonography, which didn’t cause any adverse effects or symptoms. Ultrasonography showed that the thrombus resolved spontaneously within 3 days, leaving no trace on contrast CT on the 5th postoperative day. This was considered as a thromboembolism event, that the thrombus had fallen off from the splenic vein stump and stayed in left portal vein transiently. No other postoperative complications or postsplenectomy complications were noticed during follow-ups in all patients.

## Discussion

Wandering spleen is a spleen with hypermobility which can migrate to any area of the abdomen or pelvis [11]. It’s generally considered a congenital abnormality in children and rarely encountered [5, 12, 13]. The normal spleen is located at left hypochondrium and fixed to the retroperitoneum by some main splenic suspensory ligaments, namely gastrosplenic, splenorenal, splenocolic, and splenophrenic ligaments [14, 15]. During embryogenesis, failure of fusion of the dorsal mesogastrium to the posterior abdominal wall causes absence of these suspensory ligaments and elongation of splenic vascular pedicle, leading to wandering spleen [11, 14, 15]. In children the reported female to male ratio is 2.4:1, and the female predominance is not so obvious as in adults (7:1) [4]. This was consistent with our results (2:1).

Wandering spleen is predisposed to pedicle torsion and subsequent infarction, which is the most frequent complication and main reason of symptoms [7, 13]. About 50% patients with wandering spleen are asymptomatic, but others may have acute, intermittent, or chronic symptoms due to torsion and spontaneous detorsion of the splenic pedicle [8, 14, 16]. The most common presentation was abdominal pain (93%) with a palpable abdominal mass, which was consistent with our results that all patients (6/6) presented abdominal pain/irritability, and half of them (3/6) had a palpable mass [8]. Other symptoms might include nausea, vomiting, fever, constipation, urinary symptoms, bleeding from gastric varices, gastric torsion, and hypersplenism [4].

In a recent systemic review, Bough et al. reported 82% patients with splenic torsion received splenectomy [4]. Early diagnosis and timely surgical intervention are necessary to preserve the spleen, but delayed diagnosis or misdiagnosis frequently occurs due to the rarity of the disease and the nonspecific symptoms [7, 17, 18]. In our case series the symptom duration before correct diagnosis ranged from 5 days to 12 months, and most patients had previous misdiagnoses and delayed diagnosis.

Imaging study is crucial for early and correct preoperative diagnosis [10, 19]. The rate of correct preoperative diagnosis is increasing in recent years with the advancement of imaging modalities, which has reached 61% in the 2010’s, and 80% in the 2020’s [4]. In our series no patients were correctly diagnosed before imaging study, but with imaging results all patients were correctly diagnosed before operation. Most reported cases were diagnosed by cross sectional contrast imaging, and contrast CT was the most widely used [4, 8]. Contrast CT can provide valuable and comprehensive information for diagnosis, including the location and size of the spleen, pedicle torsion (whirl sign), perfusion status, splenic vein/portal vein system thrombosis, and adjacent organ involvement (pancreatic tail torsion, stomach volvulus, compressing bladder or bowel, etc.) [10, 20]. It’s noteworthy that the whirl sign is highly specific and diagnostic for splenic pedicle torsion, which is twisted splenic pedicle intermingled with fat and/or distal pancreatic tissue, presenting as alternating circular radiodensity and radiolucency bands on contrast CT imaging [10]. And the whirl sign is better observed on dynamic image slice series, than on one still slice (see supplementary video 1). In order to make an early and correct diagnosis, we suggest that in children with both abdominal pain and abdominal mass, an early abdominal cross sectional contrast imaging (especially contrast CT) should be performed to obtain valuable diagnostic information.

Normally, the spleen sequesters one third of the total body platelets as the splenic platelet pool, which is exchangeable with peripheral circulation, playing a key role in regulating peripheral platelet counts [21]. Postsplenectomy reactive thrombocytosis is a very common phenomenon in pediatric patients, which is attributed to the loss of splenic platelet pool to sequester this portion of platelets [21, 22]. The platelet counts can start to increase by 30–100% from the 1st postoperative day on, peaking between 10–20 days postoperatively, lasting for 3–6 months and even a long term [23, 24]. In case of hypersplenism, the size of splenic platelet pool is proportional to the size of the spleen, and can sequester up to 72% of the total platelets causing thrombocytopenia [21].

In patients with splenic torsion, the splenic vein is compromised first because of its low pressure, causing splenic blood outflow obstruction while still maintaining splenic artery perfusion, which results in splenic congestion/hemorrhage and enlargement [18]. Progression of the torsion will lead to complete vein obstruction and loss of artery perfusion, which results in splenic infarction. In case of chronic mild torsion and incomplete splenic vein obstruction, increased blood outflow pressure will cause splenomegaly and hypersplenism/thrombocytopenia [25–27]. But in case of acute severe torsion with splenic infarction, the patient will be in a functional asplenia status and will have reactive thrombocytosis. In our series, four patients with preoperative thrombocytosis were all confirmed as splenic infarction by surgery and pathology, while two patients with preoperative normal platelet counts were confirmed to have viable spleens. Although in case 6 the main spleen was viable and only the accessory spleen was infarcted, the patients still had reactive thrombocytosis. This could be explained by the size of the infarcted accessory spleen almost equal to the main spleen, which means acute loss of half of the splenic platelet pool. Here we propose that preoperative thrombocytosis may predict splenic infarction in patients with splenic torsion. We also noticed this phenomenon in other published cases [17, 28].

The spleen is a main reticuloendothelial organ providing immune protection and blood filtration function (especially against encapsuled bacteria) [29]. Patients after total splenectomy have increased risks of infections (especially overwhelming postsplenectomy infection), arterial and venous thrombotic disease, pulmonary hypertension, and cancer [30]. Therefore, spleen preserving surgery should be seriously considered in patients with splenic torsion, especially in children [31]. The spleen preserving surgery generally included splenopexy and splenic autotransplantation. The former aimed to fix the viable spleen at left hypochondrium in a retroperitoneal or intrabdominal pouch with or without a mesh, and the latter aimed to implant viable splenic tissue cubes on the greater omentum [32–35]. The reported indications of splenectomy for splenic torsion included splenic infarction, splenomegaly, hypersplenism, splenic vessel thrombosis and splenic rupture [9, 36]. However, some of the indications no longer stand, since some surgical methods have been proposed to preserve the spleen in complicated situations, and the spleen has a remarkable resilience and regenerative capacity. Umeda et al. introduced a three-incision retroperitoneal pouch technique to fix a large spleen [32]. Fonseca et al. and Esposito et al. used a combined technique of partial splenectomy and splenopexy, which can be adopted in case of a partial infarcted spleen or a large spleen [37, 38]. Goyal et al. performed a salvage splenopexy for a patchy reperfused spleen after detorsion, which was proved viable after 4 months by biopsy [39]. Petroianu et al. reported a huge wandering spleen returning to normal size showed by CT 4 years after splenopexy [40]. Katsura et al. and Takayasu et al. resected large but viable wandering spleens with torsion and implanted splenic tissue to omentum, which were proved viable and functional by scintigram and absence of Howell-Jolley bodies in blood specimens [34, 35]. Bough et al. revealed that 32% of the resected wander spleen with torsion were pathologically normal or congested, and 14% had only partial or focal necrosis [4]. These spleens potentially could be preserved. Obviously, a viable wandering spleen with torsion should always be seriously consider for preservation by splenopexy or splenic autotransplantation, and splenectomy is only indicated for total splenic infarction. In our series, case 3 had a viable but large wandering spleen with torsion, which we now believe should be fixed with a three-incision retroperitoneal pouch or a mesh, or after partial splenectomy. Case 5 had a partial viable large wandering spleen with torsion and splenic vein thrombosis, which we now believe should be preserved by autotransplantation. Non-operative management of splenic torsion is not recommended, because there is a high risk of complications such as splenic infarction, rupture, infection, and intestinal obstruction, etc. But certain case was managed non-operatively with close monitoring, expecting autosplenectomy [28].

Adjacent organs involvement is common, occurring in 28% of all splenic torsions, such as pancreatic tail torsion, gastric volvulus and varices, intestinal obstruction, and urinary tract compression [4]. The failure of fusion of dorsal mesogastrium to the retroperitoneum may also cause the pancreatic tail becoming intraperitoneal, instead of retroperitoneal, which can be involved in splenic pedicle torsion [10]. Usually after detorsion and splenopexy or splenectomy, the pancreatic tail can be replaced with no further complications [6, 11]. Distal pancreatectomy should only be reserved for cases with pancreatic tail necrosis due to torsion. Gastric volvulus may also be replaced without further gastropexy [11]. Although rare, gastric varices or variceal bleeding may occur in patients with splenic torsion, and can be aggravated by the coexisting gastric volvulus, namely left-sided portal hypertension [41, 42]. This is caused by splenic vein occlusion (torsion and/or thrombosis), which leads to the blood outflow of spleen retrograde filling the short gastric veins, left gastric vein, and left gastro-epiploic vein [43]. There should be no esophageal varices in left-sided portal hypertension [42]. In case 5, pancreatic tail torsion, mescenteroaxial gastric volvulus with left-sided portal hypertension (gastric fundus varices, engorged left gastric vein and gastro-epiploic vein) coexisted. After detorsion, splenectomy, and simply replacement of pancreatic tail and stomach, no gastropexy or distal pancreatectomy were performed. The postoperative CT showed normal position of pancreas and stomach, as well as obviously relived left-sided portal hypertension.

All patients had good prognosis and no complications occurred except case 5. In case 5 a transient and asymptomatic thrombus was detected in the sagittal portion of left portal vein, which resolved spontaneously within 3 days. Since this patient had a large splenic vein thrombosis, we consider the thrombus had fallen off from the splenic vein stump causing thromboembolism. Portal vein thrombosis after splenectomy is a known complication, occurring in 0.5%-55% cases depending on different diagnostic standard and method, which generally needs anticoagulation treatment [44]. But it’s postulated that many undiagnosed asymptomatic patients were not treated with no consequences [44]. Portal vein thrombosis has been reported in patients with splenic torsion [45].We consider case 5 was not a portal vein thrombosis event, but a thromboembolism event, and since the patient was asymptomatic and the portal vein blood flow was not affected, no anticoagulation treatment was given under close ultrasonographic monitoring.

### Limitations

Due to the rarity of pediatric splenic torsion, this is just a descriptive case series with 6 patients, no statistic comparisons were allowed. Due to the retrospective nature of this study, although all patients were treated with splenectomy under certain circumstances, we cannot stress the importance of spleen preserving surgery depending on splenic viability, including splenopexy and splenic autotransplantation.

## Conclusions

In conclusion, imaging modalities are crucial to the diagnosis of pediatric splenic torsion and adjacent organ involvement. Preoperative thrombocytosis may predict splenic infarction. Spleen preserving surgery should be seriously considered over splenectomy in patients with a viable spleen.

## Supplementary Information

**Additional file 1.**

**Additional file 2.**

## Data Availability

All data generated or analyzed during this study are included in this published article and two supplementary videos.

## Abbreviations

CT: Computed tomography
MRI: Magnetic resonance imaging
